# Intraspecific Variation in the Alkaloids of *Adalia decempunctata* (Coleoptera, Coccinellidae): Sex, Reproduction and Colour Pattern Polymorphism

**DOI:** 10.1007/s10886-024-01544-4

**Published:** 2024-09-14

**Authors:** Muhammad Aslam, Oldřich Nedvěd, John J. Sloggett

**Affiliations:** 1 Maastricht Science Programme, Maastricht University, Maastricht, the Netherlands; 2 Faculty of Science, University of South Bohemia, České Budějovice, Czech Republic; 3 Institute of Entomology, Biology Centre of Czech Academy of Sciences, České Budějovice, Czech Republic

**Keywords:** Aposematism, Chemical defence, Color morph, Polymorphism, Reproductive allocation, Sexual dimorphism

## Abstract

In this paper, we examine intraspecific variation in the quantity of alkaloid chemical defence in field collected individuals of the polymorphic ladybird beetle *Adalia decempunctata* (10-spot ladybird). Like its more widely studied relative *Adalia bipunctata* (2-spot ladybird), *A. decempunctata* possesses the alkaloids adaline and adalinine, which are, respectively, the major and minor alkaloids of *A. bipunctata*. We focused especially on alkaloid concentration in relation to colour pattern morph, sex, and the relationship between female and egg parameters. There was a marked sexual dimorphism in the balance of the two alkaloids, with adaline predominating in females and adalinine predominating in males: in males, on average, over 70% of total alkaloid was adalinine. Females had a lower proportion of adalinine (< 10%) than their eggs (> 15%) and relationships between egg alkaloid and female alkaloid or fecundity were weak or non-existent. Colour pattern morph had a borderline (although not) significant relationship with adaline concentration and total alkaloid concentration, which could be further explored with laboratory reared individuals. The sexual dimorphism in alkaloid content, which seems likely due to differences in synthesis, might be related to their relative costs to the two sexes and might provide insight into the evolution of alkaloid diversity in ladybirds.

**Supplementary Information:**

The online version contains supplementary material available at 10.1007/s10886-024-01544-4.

## Introduction

In order to deter natural enemies, potential prey may be equipped with distasteful and/or toxic defensive substances that they advertise using bright colours and bold patterns to warn their enemies of danger (Eisner et al. 2007; Ruxton et al. 2018). The signal serves to enhance learning, recognition and memory (Speed 2000; Sherratt 2002), and bright displays are often adopted by animals which have well-developed chemical defences (Zvereva and Kozlov 2016). Warning colouration is a recurring theme found throughout the animal kingdom, and is particularly pronounced in some groups of insects such as ladybird beetles, which are well known for their bright colours and striking spotted patterns (e.g., Arenas et al. 2015; Ruxton et al. 2018).

The chemical defences of ladybirds are largely comprised of autogenously produced alkaloids that are secreted from the leg joints of adults and the body wall of larvae and some pupae, although they are present in all developmental stages (Brakefield 1985; Daloze et al. 1994; Aslam et al 2019). With the exception of the herbivorous Epilachninae (Sloggett 2022), ladybird chemical defences are usually comprised of one to four alkaloid types, which vary across taxa (Daloze et al 1994; Sloggett 2005). Often one alkaloid predominates alongside other minor types, which are often structurally related (e.g. Tursch et al. 1971; Lognay et al. 1996; Braekman et al. 1999).

A positive correlation has been postulated between toxicity and warning displays of aposematic animals. This phenomenon has often been observed in interspecific comparisons (e.g. Summers and Clough 2001), including in ladybirds (Arenas et al. 2015). However, inevitably there is also intraspecific defensive variation in chemically defended organisms (Speed et al. 2012). In ladybirds, such variation may be genetic (Holloway et al. 1993), but can also arise from a diversity of environmental factors such as diet, age, and parasitism (e.g. Sloggett 2022; Sakaki and Nedvěd 2023; Steele et al 2023). It is expected that such environmental variation can be correlated with intraspecific variation in warning colouration depending on resource availability. Because colour pigments could compete for compounds with anti-oxidant properties (Ahmad 1992; Griffith et al. 2006), pigment brightness can act as an honest handicap signal if these compounds are limiting, while if they are not a negative relationship between toxicity and warning signals is expected (Blount et al. 2009). In ladybirds, the former appears to be the case (e.g. Bezzerides et al. 2007; Blount et al. 2012; Wheeler et al. 2015).

The most extreme intraspecific forms of colour pattern variation in ladybirds are, however, genetic, with a number of ladybird species exhibiting strikingly different, broadly discontinuous colour-pattern genetic morphs (Majerus 1994; Sloggett and Honěk 2012). Variation in chemical defence has also been investigated in relation to this sort of colour pattern variation. No qualitative differences were found in the chemical defences of morphs of *Adalia* species over half a century ago (Tursch et al. 1973; Pasteels et al. 1973), and none have been recorded since in other polymorphic species. Additionally, biological assays have indicated that there are no notable differences in the defensive capabilities of different morphs of polymorphic species, mainly the well-studied *Harmonia axyridis* (Pallas) (Sloggett 2010; Arenas et al. 2015; Sakaki and Nedvěd 2023; Aslam and Nedvěd 2024): this would tend to indicate that quantitative differences are also absent.

**Fig. 1:**
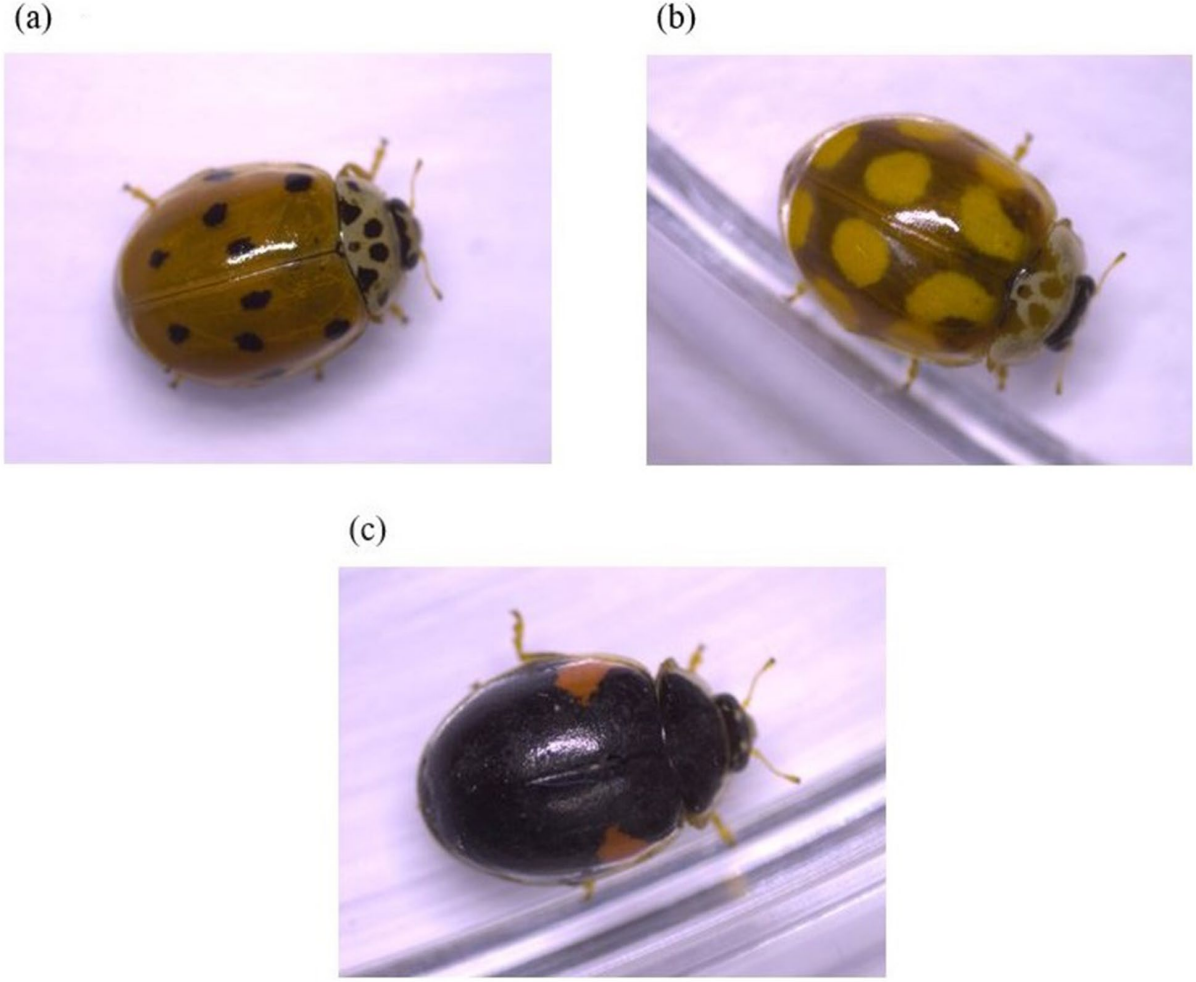
Colour pattern morph exemplars of *A. decempunctata* (after Majerus 1994): **a** typical form (= f. *typica*, see Nedvěd 2015), with 0 to 16 dark melanised spots on a yellowish to reddish background; **b** chequered form (f. *decempustulata*) with yellowish to reddish spots on a melanised brown to black background; **c** melanic form (f. *bipustulata*), with one sickle-shaped yellowish to reddish spot anteriorly on each elytron on a melanised brown to black background. The anglicized names of the forms are used throughout the paper

The ladybird *Adalia bipunctata* (L.) is one of the most well studied species in respect of both its chemical defences (e.g. de Jong et al. 1991; Marples 1993; Paul et al 2015; Oudendijk and Sloggett 2022) and polymorphism (Majerus 1994; Sloggett and Honěk 2012). *Adalia decempunctata* (L.), a more specialised species so closely related to *A. bipunctata* that the two will hybridise (Ireland et al. 1986), is much less well studied in all respects (Sloggett 2005). Like *A. bipunctata*, this species has two alkaloids, adaline and adalinine, the latter considered to be a minor alkaloid (Pasteels et al. 1973; Lognay et al. 1996). Furthermore, the species has three distinct genetic colour pattern morphs (Majerus 1994; Fig. 1). In this paper, we address variation in the alkaloids in the adults and eggs of *A. decempunctata*, including in relation to colour pattern morph, using ladybirds collected from the field.

## Materials and Methods

### Insect Material

Adults of *Adalia decempunctata* were collected in September 2018 in Maastricht, the Netherlands (50.844°N, 5.693°E). They were sexed using the abdominal criteria of Randall et al. (1992), overwintered in single sex groups in a domestic refrigerator at approximately 4 °C, and used for experiments in March 2019. At that time ladybirds were retrieved from the refrigerator and maintained in a climatically controlled cabinet at 22 °C, 70% R.H. and a 16:8 h light:dark photoperiod. They were fed with excess pea aphids, *Acyrthosiphon pisum* (Harris), a suitable aphid diet for *A. decempunctata* (e.g., Sloggett and Lorenz 2008), with a 0.5 cm^3^ piece of apple as an additional fluid source. After two weeks, ladybirds were paired at random in individual Petri dishes (9 cm diam.). Of a total of 19 pairs, most pairs produced eggs within 48 h; two pairs that failed to oviposit normally were removed from the experiment and the remaining 17 pairs that laid sufficient eggs were used. Adults included typical, chequered and melanic forms (Fig. 1). Information (i.e. sex and morph) about every individual adult male and female of all 17 numbered pairs was recorded.

### Collection of Samples for Analysis

Petri dishes were checked for eggs twice daily for 10 days after mating. If eggs were present, the adults were moved to new Petri dishes to prevent filial cannibalism. Petri dishes containing eggs were then placed in a domestic refrigerator at approximately 4 °C. Eggs were counted and frozen at -80 °C before they were collected from the Petri dishes if required for alkaloid analysis. These eggs were from those laid later in the 10-day period, as the first clutches laid by females can exhibit limited viability. Oviposition typically occurred on the sides of the Petri dish, and eggs were removed individually using a needle to manipulate them and separate them from the substrate whole. One hundred eggs per female were collected in a single sample for analysis. This sample was weighed to the nearest 0.1 mg on a Mettler Toledo XS205 balance before being refrozen at -80 °C until analysis. After the collection of eggs, males and females of all 17 pairs were separately weighed and frozen until used for extraction.

### Extraction of Alkaloid

Extraction of alkaloid followed a similar procedure used by Oudendijk and Sloggett (2022) with modifications. Each sample (adult or eggs) was homogenised in 200 µL methanol (HPLC grade) with 10 µL of a 2.5 mg/mL nicotine internal standard. After one hour, the sample was centrifuged for 5 min at 15000x*g* RCF. The supernatant containing alkaloid was removed using a micropipette and placed in a new glass tube. The liquid in the tube was dried under a flow of nitrogen to evaporate the methanol and 50 µL of chloroform was added to the tube and vortexed. Because in initial GC analyses of this 50 µL solution, there was a tendency for oversaturation of ions in some of the alkaloid GC peaks, we added 20 μL of this extract to 30 μL of chloroform and used this more diluted final product in GC analyses.

### Quantitative Analysis

GC–MS analyses were carried out on a Shimadzu GC-2010 Plus gas chromatograph with an AOC-20i autoinjector and a 2010 Ultra Mass Spectrometer. A split‐splitless injector at 200 °C and a DB5 GC column (0.25 mm diameter; 30 m length; 0.25 µm film thickness) were used. The carrier gas was helium at a constant rate of 1.05 mL min^−1^. The GC temperature program used was 60 °C for 2 min, then an increase of 40 °C min^−1^ up to 180 °C, 20 °C min^−1^ to 280 °C and 40 °C min^−1^ to 325 °C with the final temperature being held for 3 min. Mass spectra were obtained using electron ionization mode at 70 eV, scanning was done for the range m/z 35–400.

The GC peaks ladybird alkaloids and standard were identified by comparison to known mass spectra (cf. Oudendijk and Sloggett 2022). They were all eluted during the slower temperature ramp. Peaks were manually integrated to obtain peak areas. The relative amount of alkaloid in samples was calculated by comparison of the area of the alkaloid peaks to the nicotine peak as μg nicotine equivalent. Results were calculated per mg wet mass, as chemical defence concentration provides a better indication of chemical defence capability in cases, such as ladybirds, when the chemical defences are stored throughout the body (Oudendijk and Sloggett 2022).

The retention time of nicotine was approximately 6.63 min. Under the injection conditions used, both adaline and adalinine can thermally degrade and in both cases alkaloid peaks (at approximately 8.66 and 9.66 min for adaline and adalinine respectively) were accompanied by peaks of a thermally degraded product (at 7.78 and 9.05 min respectively). For these alkaloids, we added peak areas from the undegraded and degraded peaks together. There is a linear relationship between the undegraded and degraded peak areas for both alkaloids (for undegraded and degraded adaline across all 51 samples, Pearson* r* = 0.93; *P* < 0.001; for adalinine, *r* = 0.57, *P* < 0.001) supporting the view that adding the peaks together provides quantifiable results (cf. Oudendijk and Sloggett 2022).

### Statistical Analysis

Concentrations of adaline, adalinine and total measured alkaloid in adults were compared using general linear models (two-way ANOVA) with fixed factors sex and morph. Data were tested for normality and heteroscedasticity and both adaline and adalinine concentrations were log transformed [log (*y* + 1)] prior to testing. The interactive term was included in the model. We subsequently carried out a similar comparison of the proportion of adalinine in the total alkaloid measured [concentration adalinine/(concentration adaline + concentration adalinine)]. These data were logit transformed prior to testing (Warton and Hui 2011). 

Female fecundity was tested as the number of eggs laid per day over the last seven days of oviposition, as at this period all females were ovipositing. We carried out general linear model (ANCOVA) estimating the effects of fixed factor morph and covariate female size (Dixon and Guo 1993) on these fecundity data. Concentration of egg adaline and adalinine, total alkaloid and proportion adalinine were tested for a correlation with female fecundity. Each of the egg alkaloid parameters were also tested for a correlation with the same maternal alkaloid parameters. The proportion of adalinine in eggs was compared to maternal adalinine proportion in a paired t-test using logit transformed data. Adaline, adalinine (log transformed), total measured alkaloid and proportion of adalinine (logit transformed) were tested against fixed effect maternal morph using a general linear model (one-way ANOVA). No tests of eggs against paternal factors were carried out, since the wild females used in this study may have mated prior to our study.

All analyses were carried out in SPSS version 28.0.1.0. Raw data is provided in Table S1.

## Results

Both adaline and adalinine were detected in all samples. There was no significant relationship between the concentrations of adaline and adalinine in males or females, but a positive relationship was observed in eggs (Fig. S1).

### Adults

**Fig. 2:**
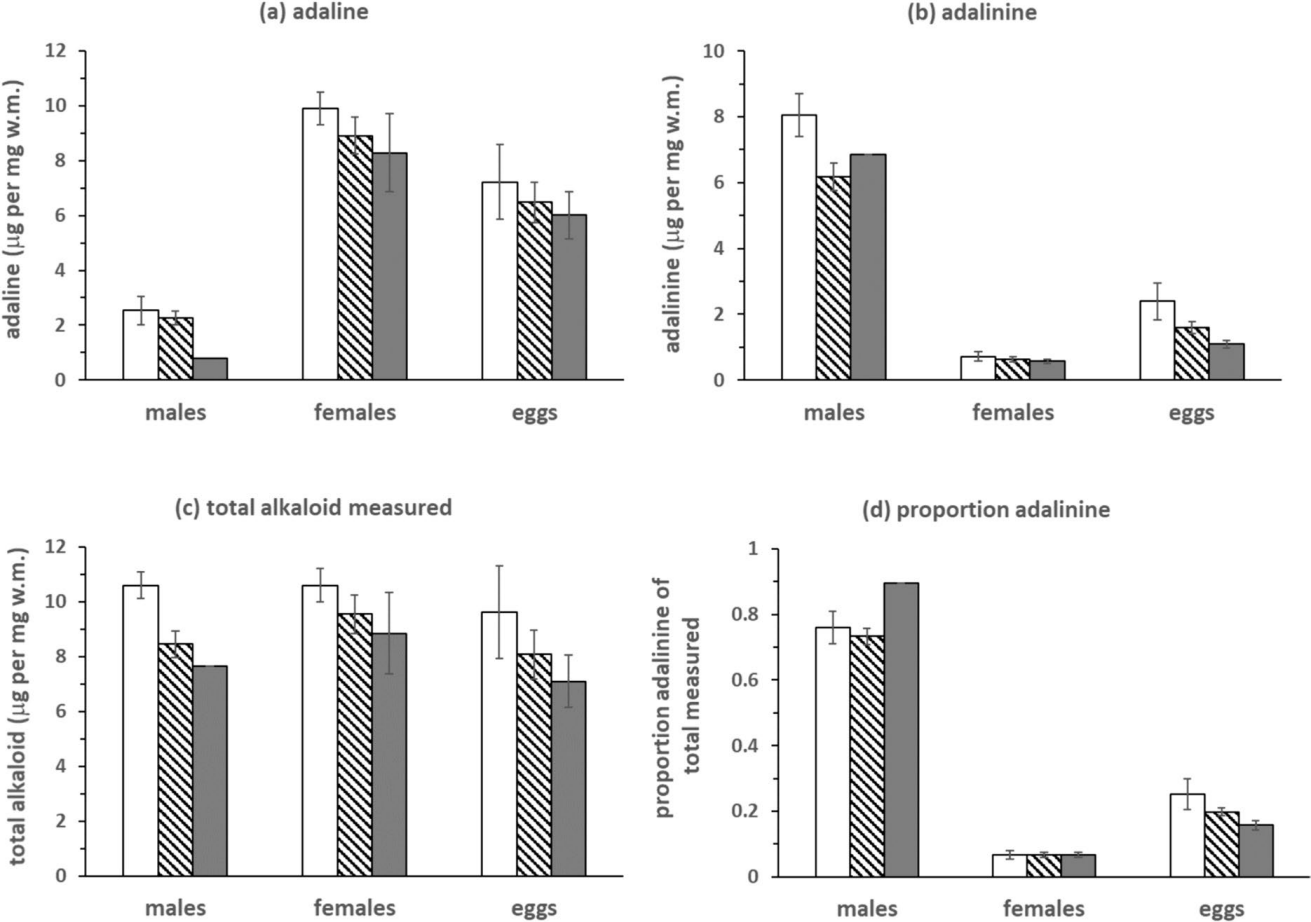
Means ± standard errors for (**a**) concentration of adaline (**b**) concentration of adalinine (**c**) total alkaloid concentration measured and (**d**) proportion adalinine of total alkaloid across the three morphs of male adults, female adults and maternal morphs of eggs. White bars = typical (*n* = 5 for males, *n* = 6 for females/eggs); striped bars = chequered (male, *n* = 11; female/eggs, *n* = 6); dark bars = melanic (male, *n* = 1, female/egg, *n* = 5)

There was a strikingly significant difference in the amounts of adaline and adalinine in males and females, with females having more adaline and males having more adalinine (Table 1; Fig. 2a and b). Interestingly there was no difference in the total alkaloid measured (Table 1; Fig. 2c), despite its different composition with a significantly higher proportion of adalinine (of total alkaloid) in males (Table 1; Fig. 2d).

**Table 1:** Results of general linear models on adult data, for sex, morph and the interaction for the individual *A. decempunctata* alkaloids, total alkaloid measured and the proportion adalinine of the total

	F	df	P
ADALINE^a^			
sex	**114.16**	**1**	** < 0.001**
colour	3.08	2	0.06
sex*colour	1.01	2	0.38
ADALININE^a^			
sex	**465.43**	**1**	** < 0.001**
colour	2.28	2	0.12
sex*colour	1.29	2	0.29
TOTAL ALKALOID MEASURED			
sex	0.79	1	0.38
colour	3.04	2	0.06
sex*colour	0.29	2	0.75
PROPORTION ADALININE/TOTAL ALKALOID MEASURED^b^			
sex	**428.50**	**1**	** < 0.001**
colour	2.06	2	0.15
sex*colour	2.02	2	0.15

^a^data transformed log(y + 1) ^b^data logit transformed

There was a non-significant trend (p = 0.06) towards a colour pattern difference for adaline, with the typical form having the most and the melanic form having the least. The pattern was repeated in both sexes, as well as in eggs (although these also showed no significance, see below) (Table 1; Fig. 2a). For adalinine, the more non-significant differences were also not consistent between the sexes (Table 1; Fig. 2b). When the alkaloids were combined to give total alkaloid measured, the differences measured for adaline gave the overall measurements the same pattern and a similar non-significant trend (also *P* = 0.06; Table 1; Fig. 2c). There was no relationship between morph and adalinine as a proportion of total alkaloid (Table 1; Fig. 2d).

### Eggs

**Fig. 3:**
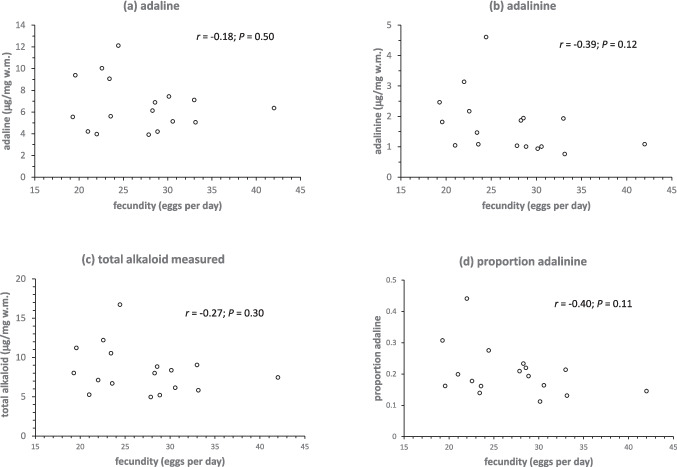
**a** Adaline concentration (**b**) adalinine concentration (**c**) total alkaloid concentration measured and (**d**) proportion of adalinine, all for eggs correlated to maternal fecundity

Neither female size nor morph had a significant effect on fecundity (size, *F*_1,11_ = 1.96; *P* = 0.19; morph F_2,11_ = 0.81, *P* = 0.47) nor was the interaction between them significant (*F*_2,11_ = 1.17, *P* = 0.35). None of the egg alkaloid parameters (adaline, adalinine, total, proportion adalinine) exhibited a significant correlation with maternal fecundity, although all relationships were negative (Fig. 3a-d).

**Fig. 4:**
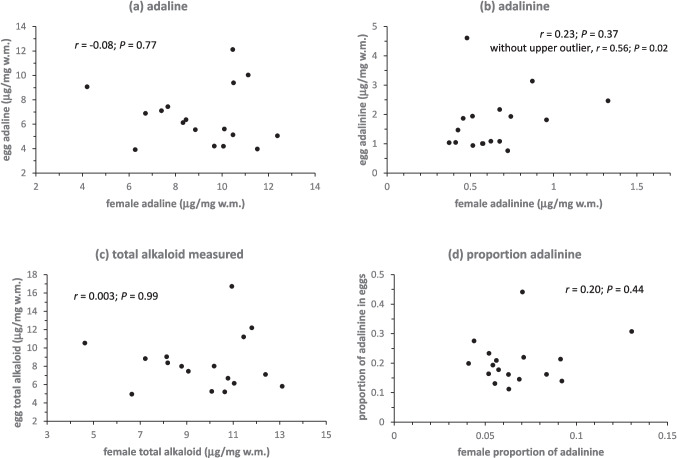
**a** Adaline concentration (**b**) adalinine concentration (**c**) total alkaloid concentration measured and (**d**) proportion of adalinine, female-egg correlations. For adalinine (**b**), a second correlation was carried out excluding the outlier with the highest adalinine value for eggs

Egg alkaloid levels were also not well correlated with maternal alkaloid for either alkaloid or the total alkaloid measured (Fig. 4a-c), except questionably, adalinine when an outlier point was removed, which was then positively correlated (Fig. 4b). Adalinine as a proportion of the total alkaloid measured was also not correlated with maternal adalinine proportion (Fig. 4d), although the proportion of adalinine was, however, higher in eggs than in the females from which they came in all 17 cases (paired t-test on logit transformed data, *t* = 10.10, 16 *d.f*., *P* < 0.001).

There were no significant differences in the relative amount of adaline, across eggs from different female morphs (One-way ANOVA, *F*_2,14_ = 0.34, *P* = 0.72; Fig. 2a), as was also the case for adalinine (One-way ANOVA on log transformed data *F*_2,14_ = 3.20, *P* = 0.08; Fig. 2b) and total alkaloid measured (One-way ANOVA, *F*_2,14_ = 0.98, *P* = 0.40; Fig. 2c). The proportion of adalinine in eggs did not vary across eggs from different female morphs (One-way ANOVA on logit transformed data *F*_2,14_ = 2.14, *P* = 0.15; Fig. 2d).

## Discussion

The most notable aspect of our results is the apparently sexually dimorphic occurrence of the two alkaloids in *A. decempunctata*. Adalinine is a minor alkaloid of *A. bipunctata* (Lognay et al. 1996; Oudendijk and Sloggett 2022), apparently in all life history stages (Lognay et al. 1996; J.J. Sloggett unpub. data). Adalinine is also already known from adult *A. decempunctata* (Lognay et al. 1996) but the abundance described here in males makes it the dominant alkaloid and much more abundant than in females or eggs. Such quantitative dimorphism in the relative amounts of different alkaloids has not been described in ladybirds before, although in general females tend to have higher concentrations of alkaloids than males, including in *A. bipunctata* (Holloway et al. 1991, 1993; de Jong et al. 1991, but see Oudendijk and Sloggett 2022).

This might in part be related to female egg production: in *A. bipunctata*, after reflex bleeding females accumulate alkaloid more slowly than males, probably because they allocate alkaloids to eggs (Oudendijk and Sloggett 2022). If, as appears to be the case here, females allocate proportionately more adalinine to eggs than they have themselves, but both sexes synthesize the two alkaloids at the same rate, then ultimately males could end up with a higher proportion of adalinine in their bodies. However, no such marked phenomenon has been observed in *A. bipunctata* (Oudendijk and Sloggett 2022) and it is hard to envisage such a large difference developing by solely by these means, suggesting that the dimorphism more likely occurs predominantly through differences in synthesis.

It appears that adalinine is synthesized from adaline in *Adalia* spp. (Laurent et al. 2001): if this is the case, then adalinine must be more energetically costly to produce due to the additional synthetic steps. Possibly males invest more in this defensive molecule than females due to the latter’s high reproductive costs. Males could even transfer adalinine to females as a nuptial gift, given its higher occurrence in eggs: alkaloid transfer from male to female is known in the ladybird *Epilachna paenulata* (Camarano et al. 2009). Alternatively, the costs of storing adalinine could be lower if, for example, adaline presents a serious risk of self-poisoning: this synthesis of a less toxic storage compound is known from other chemically defended organisms (e.g. Hartmann 2004; Brückner et al. 2017).

The sexual difference in the abundance of the two alkaloids in *A. decempunctata* poses a potential issue in understanding the role of colour pattern in signalling defensive capability. A weak, borderline significant relationship (*P* = 0.06) exists for adaline and total alkaloid. Bearing in mind that this is non-significant, this could be a consequence of our relatively small sample sizes, which also vary across morph in the two sexes. A non-significant result could be exacerbated by the fact that all morphs of field-collected adults will exhibit alkaloid variation in response to a diversity of factors. These include diet, reflex bleeding, age, temperature and parasite infection (e.g. de Jong et al. 1991; Steele et al. 2020, 2023; Oudendijk and Sloggett 2022; Sakaki and Nedvěd 2023). A laboratory study could eliminate this variation. However, in the absence of an understanding of the roles of the two alkaloids, it is hard from the perspective of a predator to interpret whether the different colour pattern morphs really convey any information about relative distastefulness and toxicity. It should also be borne in mind that many other factors play a role in colour pattern polymorphism apart from chemical defence (Sloggett and Honěk 2012; Briolat et al. 2019).

There was no clear relationship between maternal alkaloid and egg alkaloid in this study, except possibly for adalinine, where the relationship was positive. Paul et al. (2015, 2018) reported contradictory results for adaline: in *A. bipunctata* in their experiments: in one study, they found no correlation, whereas in another there was a positive relationship. In this study of *A. decempunctata*, there was a tendency for alkaloids to decline with female fecundity, though not significantly. Data on adaline in *A. bipunctata* is also contradictory on this point (Paul et al. 2015, 2018) but in one case, they also observed a negative relationship (Paul et al. 2018). In both studies of *A. bipunctata*, adalinine was not quantified, making a direct comparison with our study difficult, but if a relationship, albeit weak, with female parameters exists, for adaline in *A. bipunctata* and adalinine (but not adaline) in *A. decempunctata*, then this might reflect the relative importance of the alkaloids in the two species.

Ladybirds display a high taxon-related alkaloid diversity (Daloze et al. 1994; King and Meinwald 1996; Laurent et al. 2005) and it is not yet clear why such diversity exists. Presumably, new major alkaloids evolve initially as minor components. The alkaloids of only two *Adalia* species have been identified: however, cautiously assuming that adaline as the major and adalinine as the minor alkaloid is the ancestral condition, the altered situation in *A. decempunctata* could throw light on how changes in chemical defences evolve in taxa such as ladybirds, where autogenous production predominates.

## Supplementary Information

Below is the link to the electronic supplementary material.

Supplementary file1 (PDF 449 KB)

Supplementary file2 (PDF 206 KB)

## Data Availability

Data is provided in the supplementary materials.
