# Supplementary material for: Intraspecific Variation in the Alkaloids of Adalia decempunctata (Coleoptera, Coccinellidae): Sex, Reproduction and Colour Pattern Polymorphism
Source: J Chem Ecol. 2024 Sep 14;50(11):790–8. doi: 10.1007/s10886-024-01544-4 (PMC11543752; doi:10.1007/s10886-024-01544-4)
Supplement: Supplementary file 1 — Supplementary file1 (PDF 449 KB) [file 10886_2024_1544_MOESM1_ESM.pdf]

Table S1: raw data and calculated parameters for our experiments. Note:alkaloid measured as µg nicotine equivalent

Pair No.	Morph	Sex	Mass (mg)	Egg number per day	Area of nicotene GC peak	Area of adaline GC peak	Area of degraded adaline GC peak	Area of adalinine GC peak	Area of degraded adalinine GC peak	Total adaline peak area	Total adalinine peak area	Relative adaline (µg)	Relative adalinine (µg)	adaline per unit mass (µg/mg)	adalinine per unit mass (mg/mg)	Total alkaloid per unit mass (µg/mg)	ratio adalinine /total
1	Chequered	Male	10.7		7593905	9918076	295472	8674247	13318737	10213548	21992984	33.62416	72.40341	3.142445	6.766674	9.909118766	0.682873
2	Chequered	Male	7.4		10094659	4750445	35435	3497874	11928443	4785880	15426317	11.85251	38.20416	1.60169	5.162724	6.764413669	0.763218
3	Chequered	Male	9.3		9851948	5866264	342802	5335289	10885941	6209066	16221230	15.75593	41.16249	1.694187	4.426075	6.120261153	0.723184
4	Chequered	Male	9.4		12812432	15161919	284318	10577043	18171731	15446237	28748774	30.13916	56.09547	3.206294	5.967603	9.173896328	0.650498
6	Chequered	Male	9.3		6996119	2774283	222045	7153200	9081708	2996328	16234908	10.70711	58.01398	1.151302	6.238062	7.389364156	0.844195
7	Chequered	Male	8.4		10221077	3058086	275511	14283551	15867741	3333597	30151292	8.153732	73.74783	0.970682	8.779504	9.750186574	0.900445
8	Chequered	Male	9		5647251	5487571	270947	5134765	4786972	5758518	9921737	25.49257	43.92286	2.832508	4.880318	7.71282592	0.632754
10	Typical	Male	9.3		9023037	11659659	340724	10646014	10704014	12000383	21350028	33.24929	59.15422	3.575192	6.360669	9.935861116	0.640173
11	Chequered	Male	5.7		7058051	5045041	189550	6271606	5204867	5234591	11476473	18.54121	40.65029	3.252843	7.13163	10.38447304	0.686759
12	Typical	Male	7.6		7306649	5125653	193403	11699663	9920176	5319056	21619839	18.19937	73.97317	2.394654	9.733312	12.12796539	0.802551
13	Typical	Male	9.7		5136832	7303944	273160	7854689	6366415	7577104	14221104	36.87635	69.21145	3.801685	7.135201	10.93688648	0.652398
14	Chequered	Male	9.6		8722154	9537276	265286	12977577	8495288	9802562	21472865	28.09674	61.54691	2.926743	6.411136	9.337879666	0.686573
15	Melanic	Male	7.2		10861794	2309776	200132	13834143	7612715	2509908	21446858	5.776919	49.36307	0.80235	6.855981	7.658331145	0.895232
16	Typical	Male	7.8		2718736	969869	104390	5406194	2623772	1074259	8029966	9.878295	73.83915	1.266448	9.466557	10.73300546	0.882004
17	Chequered	Male	9.5		8073383	5009002	168611	9486318	4419938	5177613	13906256	16.03297	43.06205	1.687681	4.532847	6.220528358	0.728692
19	Chequered	Male	11.3		5356188	5613133	206057	13071544	5665109	5819190	18736653	27.16106	87.4533	2.403634	7.73923	10.14286397	0.763022
20	Typical	Male	8.4		7130717	3717349	199225	13365419	4820451	3916574	18185870	13.73135	63.75891	1.634684	7.590347	9.225030713	0.822799
1	Typical	Female	13.2	22	7045310	41907417	935527	2563151	681690	42842944	3244841	152.0265	11.51419	11.51716	0.872287	12.3894437	0.070406
2	Melanic	Female	14.3	42	5709358	26737060	859635	1632770	401637	27596695	2034407	120.8397	8.908213	8.450332	0.622952	9.073283874	0.068658
3	Typical	Female	11.4	30.14286	6512467	22072938	704413	1253549	275654	22777351	1529203	87.43749	5.870291	7.669955	0.514938	8.184893143	0.062913
4	Melanic	Female	12.2	23.57143	7052195	33794977	962541	1987154	338598	34757518	2325752	123.2152	8.244781	10.09961	0.675802	10.77541211	0.062717
6	Melanic	Female	12.9	27.85714	10610828	33105815	1203954	1735941	302868	34309769	2038809	80.83669	4.803605	6.26641	0.372372	6.63878263	0.05609
7	Typical	Female	10.7	22.57143	5708967	26124899	1055641	1411651	239698	27180540	1651349	119.0256	7.231383	11.12389	0.67583	11.79972255	0.057275
8	Melanic	Female	9.3	23.42857	8664288	12948326	574323	1174076	196464	13522649	1370540	39.01835	3.954566	4.195522	0.425222	4.620743713	0.092025
10	Melanic	Female	17.2	33.14286	7094682	58283001	2153480	3114785	420719	60436481	3535504	212.964	12.45829	12.38163	0.724319	13.10594842	0.055266
11	Typical	Female	9.9	24.42857	5946345	23591993	1039842	972033	156888	24631835	1128921	103.5587	4.746281	10.46048	0.479422	10.93989907	0.043823
12	Chequered	Female	11	19.57143	6295026	27883760	1177102	2380845	270373	29060862	2651218	115.412	10.52902	10.492	0.957184	11.4491837	0.083603
13	Typical	Female	13.5	19.28571	3916013	17934908	782108	2535429	270028	18717016	2805457	119.4903	17.91016	8.85113	1.326679	10.17780878	0.13035
14	Chequered	Female	13.3	33	4631638	17508383	713024	1540254	290412	18221407	1830666	98.35293	9.88131	7.394957	0.742956	8.137912881	0.091296
15	Chequered	Female	13.1	28.57143	9913584	33962276	868302	2122954	541265	34830578	2664219	87.83548	6.718607	6.704999	0.512871	7.217869552	0.071056
16	Typical	Female	9.2	21	884705	2910276	236501	133915	0	3146777	133915	88.92165	3.784171	9.665396	0.411323	10.07671926	0.040819
17	Chequered	Female	11.1	28.28571	7005576	25082103	806330	1139633	280593	25888433	1420226	92.3851	5.068199	8.322982	0.456594	8.77957626	0.052006
19	Chequered	Female	12.7	28.85714	7366135	36346604	1296243	1874168	280937	37642847	2155105	127.7564	7.314233	10.05956	0.575924	10.63548594	0.054151
20	Chequered	Female	11.2	30.57143	7571145	34206524	1310762	1671428	269657	35517286	1941085	117.2784	6.409483	10.47129	0.572275	11.04356544	0.05182
1	Typical	Egg	11.7	22	32939467	60318417	918248	36687950	11690902	61236665	48378852	46.47667	36.718	3.972365	3.13829	7.110655135	0.44135
2	Melanic	Egg	10.9	42	4630828	12420683	440084	1999586	190803	12860767	2190389	69.43017	11.82504	6.36974	1.084866	7.45460639	0.14553
3	Typical	Egg	10.7	30.14286	37741395	117623963	2493502	12675812	2501368	120117465	15177180	79.56613	10.0534	7.436087	0.93957	8.375657117	0.112179
4	Melanic	Egg	9.8	23.57143	30480526	65424334	1608308	11148286	1781438	67032642	12929724	54.97989	10.60491	5.610193	1.082133	6.692326358	0.161698
6	Melanic	Egg	13.9	27.85714	1877244	3843407	247671	938969	143414	4091078	1082383	54.4825	14.41452	3.919604	1.037016	4.956620435	0.209218
7	Typical	Egg	10.9	22.57143	13691621	58555894	1322642	11123031	1823076	59878536	12946107	109.3343	23.63874	10.03067	2.168692	12.19935884	0.177771
8	Melanic	Egg	10.8	23.42857	26371971	100987832	2299019	14329331	2404082	103286851	16733413	97.91347	15.86288	9.066062	1.468785	10.53484721	0.139422
10	Melanic	Egg	13.2	33.14286	27722593	71989097	2010077	9819460	1333934	73999174	11153394	66.73183	10.05804	5.055442	0.761972	5.817414086	0.130981
11	Typical	Egg	10.8	24.42857	6667102	34008173	889484	11910673	1361070	34897657	13271743	130.8577	49.76579	12.11645	4.607943	16.72439401	0.275522
12	Chequered	Egg	10.5	19.57143	6358544	24788605	295308	3648915	1200407	25083913	4849322	98.62286	19.06617	9.392654	1.815825	11.20847904	0.162005
13	Typical	Egg	10.4	19.28571	9461728	21214154	641063	8708946	995987	21855217	9704933	57.74637	25.6426	5.552535	2.465635	8.018170169	0.307506
14	Chequered	Egg	10.2	33	8019830	22596471	661126	5705162	613894	23257597	6319056	72.50028	19.69822	7.107871	1.931198	9.039068979	0.21365
15	Chequered	Egg	11	28.57143	5000821	14640361	521010	3864475	408292	15161371	4272767	75.79441	21.36033	6.890401	1.941848	8.832248836	0.219859
16	Typical	Egg	10.3	21	7274453	12167102	446402	2832350	300670	12613504	3133020	43.34863	10.7672	4.208605	1.045359	5.253964586	0.198966
17	Chequered	Egg	11.6	28.28571	8556391	23639586	705402	6656456	751938	24344988	7408394	71.13101	21.64579	6.131983	1.866016	7.997999733	0.23331
19	Chequered	Egg	10.2	28.85714	4492904	7402331	290700	1641038	203085	7693031	1844123	42.80656	10.26131	4.196722	1.006011	5.202732453	0.193362
20	Chequered	Egg	12.3	30.57143	8566946	20954541	680721	3847538	396651	21635262	4244189	63.13587	12.38536	5.132997	1.00694	6.139937168	0.163998
