# Supplementary material for: Intraspecific Variation in the Alkaloids of Adalia decempunctata (Coleoptera, Coccinellidae): Sex, Reproduction and Colour Pattern Polymorphism
Source: J Chem Ecol. 2024 Sep 14;50(11):790–8. doi: 10.1007/s10886-024-01544-4 (PMC11543752; doi:10.1007/s10886-024-01544-4)

FIGURE S1: Relationship between concentrations of adaline and adalinine in (a) males, (b) females and (c) eggs

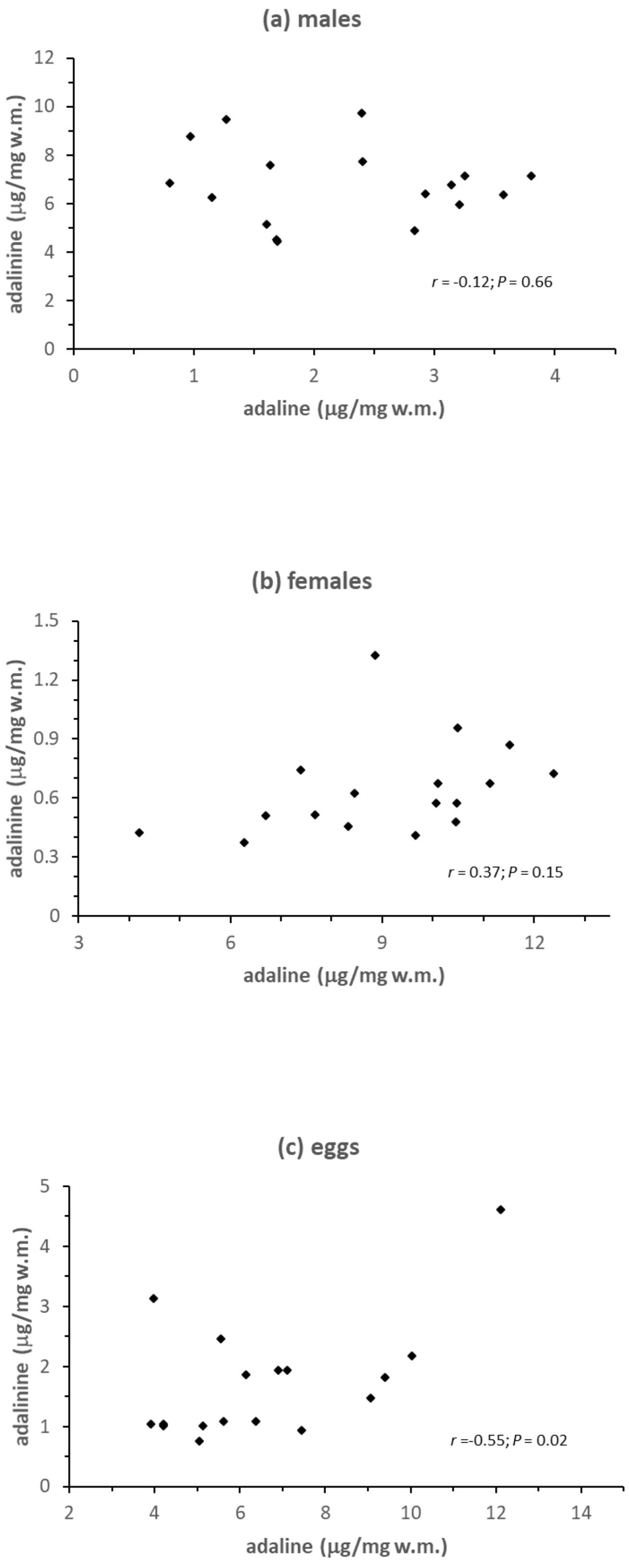

Supplement: Supplementary file 2 — Supplementary file2 (PDF 206 KB) [file 10886_2024_1544_MOESM2_ESM.pdf]
